# Supplementary material for: Perioperative outcomes and hospitalization costs of radical vs. conservative surgery for hepatic cystic echinococcosis: A retrospective study
Source: PLoS Negl Trop Dis. 2024 Nov 13;18(11):e0012620. doi: 10.1371/journal.pntd.0012620 (PMC11559981; doi:10.1371/journal.pntd.0012620)
Supplement: S1 Text — (DOCX) [file pntd.0012620.s002.docx]

**Age (years)**: the number of years since birth.

**Gender (male/female)**: the biological sex of the patient.

**Cyst location (Left lobe/right lobe/ both the lobes)**: the location of the hydatid cyst in patients from abdominal imaging.

**Cyst diameter (cm)**: the diameter of the hydatid cyst from abdominal ultrasound.

**Number of cysts**: the number of the hydatid cyst in patients from abdominal imaging.

**WHO cyst classification (CE1, CE2, CE3a, CE3b, CE4, CE5)**: classification according to the World Health Organization's criteria.

**Epigastric pain**: presence of pain in the upper abdominal region.

**Abdominal mass**: presence of a palpable mass in the abdomen.

**Fever**: body temperature greater than 38.0°C.

**Extrahepatic cyst**: presence of extrahepatic hydatid cyst, such as pulmonary or abdominal hydatid cyst.

**Open abdominal surgery**: traditional procedure involving a large incision, as opposed to laparoscopic surgery.

**Operative time (min)**: duration of the surgical procedure.

**Blood loss (ml)**: the amount of blood lost during the surgery.

**Blood transfusion (ml)**: the volume of blood transfused during the surgery.

**Overall morbidity (yes/no)**: including bile leak, effusion (postoperative pleural or abdominal effusion), pulmonary infection, death, ICU stay, abdominal drainage time days>7, incision infection, intestinal obstruction.

**Death (yes/no):** mortality occurring during or after the surgery.

**Bile leak (yes/no)**: postoperative drainage fluid is yellow or dark green bile.

**Effusion (yes/no)**: postoperative pleural or abdominal effusion

**Pulmonary infection (yes/no)**: infection of the lungs occurring after the surgery.

**ICU stay (yes/no)**: duration of stay in the Intensive Care Unit postoperatively.

**Abdominal drainage time (≤7/＞7)**: duration for which the abdominal drain is in place postoperatively.

**Postoperative hospital stay (days)**: days of postoperative hospital stay.

**Incision infection**: infection at the site of the surgical incision.

**Intestinal obstruction**: blockage of the intestines occurring after the surgery.

**Total cost**: derived from the patient’s admission records, including all expenses incurred during the hospital stay. Total cost included surgery-related costs, non-surgery-related costs, consumables costs, medication costs, examination costs, and other costs (Total).

**Surgery-related costs** included surgical treatment costs, anesthesia costs, and surgery fees.

**Non-surgery-related costs** consisted of treatment operation costs.

**Consumables costs** encompassed examination material costs, treatment material costs, and surgical material costs.

**Medication costs** were comprised of Traditional Chinese Medicine (TCM) treatment costs, cistern medicine costs, antibiotic costs, Chinese patent medicine costs, blood transfusion costs, albumin product costs, and coagulation factor product costs. **Examination costs** included pathology diagnosis costs, laboratory diagnosis costs, imaging diagnosis costs, and clinical diagnosis costs.

**Other costs (Total)** consisted of medical service fees, nursing fees, rehabilitation costs, and specific additional costs.
