# Supplementary material for: Perioperative outcomes and hospitalization costs of radical vs. conservative surgery for hepatic cystic echinococcosis: A retrospective study
Source: PLoS Negl Trop Dis. 2024 Nov 13;18(11):e0012620. doi: 10.1371/journal.pntd.0012620 (PMC11559981; doi:10.1371/journal.pntd.0012620)
Supplement: S2 Text — (DOC) [file pntd.0012620.s003.doc]

**STROBE Statement—Checklist of items that should be included in reports of cohort studies**

**1. (*a*) Indicate the study’s design with a commonly used term in the title or the abstract**

Page 1. Title: “Perioperative outcomes and hospitalization costs of radical vs. conservative surgery for hepatic cystic echinococcosis: A retrospective study”

Page 3. Abstract Method Section: A retrospective cohort study was conducted on patients undergoing surgical treatment at the First People's Hospital of Kashi Prefecture from July 1, 2012, to October 1, 2023.

**(b) Provide in the abstract an informative and balanced summary of what was done and what was found**

Page 3. Abstract: Method (A retrospective cohort study was conducted on patients undergoing surgical treatment at the First People's Hospital of Kashi Prefecture from July 1, 2012, to October 1, 2023.)

Page 3-Abstract: Result (Of the 434 patients included, 324 underwent conservative surgery and 110 underwent radical surgery. After propensity score-matching, 182 conservative surgery and 102 radical surgery patients were compared. Radical surgery patients experienced longer operative time, higher intraoperative blood loss, increased blood transfusion, and higher costs compared to conservative surgery patients. However, no differences were observed in short-term outcomes, including overall morbidity, death, bile leak, effusion, pulmonary infection, incision infection, intestinal obstruction, ICU stay, abdominal drainage time, and postoperative hospital stays.)

Page 3-4. Abstract: Conclusion (This study suggests that radical surgery is associated with greater surgical complexity and higher hospitalization costs, while it doesn’t offer a significant short-term advantage. Conservative surgery may be a viable option in resource-limited settings or for patients unsuitable for complex procedures. Further research with long-term follow-up is needed to determine the optimal approach.)

**2. Explain the scientific background and rationale for the investigation being reported**

Page 4-5.

**Scientific Background**:

Disease Background: The introduction begins by explaining that cystic echinococcosis (CE) is a zoonotic infectious disease caused by Echinococcus granulosus, with the liver being the most affected organ. This provides the fundamental information and significance of the disease.

Clinical Impact: It further describes that large cyst may lead to serious complications, such as cystobiliary communication and acute cholangitis, and that cyst ruptures in CE can induce symptoms such as urticaria, fever, and even anaphylactic shock, and may also lead to the dissemination of the parasite. This highlights the severity and urgency of addressing the disease.

Economic Burden: The introduction mentions that the global annual expenditure on CE is estimated to exceed 760 million US dollars, emphasizing the economic burden in endemic regions.

**Rationale for the Investigation:**

Treatment Methods: It explains that Management of hepatic cystic echinococcosis (HCE) is complex and includes surgical treatments, percutaneous treatment, chemotherapy, and “watch and wait”. The introduction details the categorization of surgical treatments into radical surgery (RS) and conservative surgery (CS). RS, such as hepatectomy and total cystectomy, offers the advantage of completely removing the lesion with a low risk of recurrence, but it is more complex and traumatic compared to CS, like sub-total cystectomy and partial cystectomy.

**Controversy and Research Gaps:** The introduction highlights that significant controversy still exists regarding the intraoperative and short-term postoperative outcomes for RS and CS, and that there is limited research on the associated costs. Many studies are limited by factors insufficient adjustment for confounding biases.

**Regional Context:** It notes that Kashi, located in the western part of China, is an endemic area for HCE and has considerable experience in the standardized diagnosis and treatment of the disease.

**3. State specific objectives, including any prespecified hypotheses**

Page 5-6. “This study aims to provide a real-world comparison of the perioperative outcomes and hospitalization costs associated with radical and conservative surgery to the management of HCE. We hypothesize that RS, compared to CS, will result in higher risks and costs without significant differences in short-term perioperative outcomes based on our experience.”

**4. Present key elements of study design early in the paper**

Page 6. “A retrospective analysis was conducted on the clinical data of patients who underwent surgical treatment for HCE in the Department of Hepatobiliary and Pancreatic Surgery, the First People's Hospital of Kashi Prefecture from July 1, 2012, to October 1, 2023.”

**5. Describe the setting, locations, and relevant dates, including periods of recruitment, exposure, follow-up, and data collection**

Page 6. “A retrospective analysis was conducted on the clinical data of patients who underwent surgical treatment for HCE in the Department of Hepatobiliary and Pancreatic Surgery, the First People's Hospital of Kashi Prefecture from July 1, 2012, to October 1, 2023.”

Page 6. “We collected clinical data from patients’ hospital records from the medical record system. Patients were divided into RS group and CS group based on the surgical method performed by the patients.”

**6.(a) Give the eligibility criteria, and the sources and methods of selection of participants. Describe methods of follow-up**

Page 6. “Inclusion criteria were patients diagnosed with HCE based on clinical history, physical examination, and imaging (Color Doppler Ultrasound or CT), who subsequently underwent surgical treatment. Patients with incomplete medical records (specifically missing preoperative Color Doppler Ultrasound results, surgical records, or postoperative clinical course records) were excluded.”

In this study, no follow-up was planned or conducted as the focus was on the immediate postoperative outcomes. Data were collected on short-term results after surgical intervention without subsequent tracking or additional follow-up assessments. This design was chosen because the primary interest was in evaluating the immediate effects of the treatment rather than long-term outcomes.

**(b) For matched studies, give matching criteria and number of exposed and unexposed**

Page 8. Propensity Score Matching was utilized to balance covariates between RS and CS groups, using demographic and clinical characteristics. We performed 1:2 greedy nearest neighbor matching with a caliper of 0.1. Specific numbers of matched exposed and unexposed participants will be detailed in the results section.

**7. Clearly define all outcomes, exposures, predictors, potential confounders, and effect modifiers. Give diagnostic criteria, if applicable**

Detailed definitions of the indicators can be found in S2.

**8. For each variable of interest, give sources of data and details of methods of assessment (measurement). Describe comparability of assessment methods if there is more than one group**

Page 6. “We collected clinical data from patients’ hospital records from the medical record system.”; “Detailed definitions are detailed in S2”

Page 5 “These indicators are also the confounding factors adjusted for during the Propensity Score Matching (PSM) process. "; The comparability of outcome measures across groups is ensured as we have employed Propensity Score Matching PSM to reduce confounding bias.”

**9. Describe any efforts to address potential sources of bias**

Page 8. “To mitigate the impact of potential confounders, we utilized PSM a statistical method designed to equalize covariates across treatment groups. This ensures that differences observed in outcomes are more likely attributed to the treatments rather than confounding factors.”

**10. Explain how the study size was arrived at**

Page 10. Due to the inability to accurately estimate the surgical situation in this region, traditional sample size calculation was not performed for this study. However, to evaluate the statistical validity of our results, we calculated the statistical power in the subsequent analysis.

**11. Explain how quantitative variables were handled in the analyses. If applicable, describe which groupings were chosen and why**

Page 7-8. In our manuscript, we described the grouping criteria and reasons for 'the number of cysts' and 'abdominal drainage time.' The number of cysts was divided into two groups: equal to 1 and greater than 1 because single and multiple cysts impact study outcomes differently. Abdominal drainage time was divided into two groups: less than or equal to 7 days and greater than 7 days, based on clinical observations, representing different rates of postoperative recovery.

**12. (a) Describe all statistical methods, including those used to control for confounding**

Page 8-9. We described all statistical methods in the “Statistical analysis”.

**(b) Describe any methods used to examine subgroups and interactions**

This study divided hepatic cystic echinococcosis patients into RS and CS groups for a direct comparison of short-term outcomes. The focus was on assessing each group's response in terms of operative duration, blood loss, transfusion volumes, and postoperative metrics like complications and hospital stays. This analysis is critical for understanding the impact of each surgical approach on patient recovery.

Our study aimed to assess the efficacy of RS versus CS without exploring interaction effects between surgical types and variables such as patient demographics or cyst characteristics. We focused on providing straightforward comparisons between the two surgical methods, ensuring that our conclusions directly reflect the effectiveness of these approaches without interference from other modifying factors.

**(c) Explain how missing data were addressed**

Page 8. “In our analysis, three individuals were excluded due to extensive missing data, which precluded meaningful analysis. No missing data issues were observed in the remaining subjects.”

**(d) If applicable, explain how loss to follow-up was addressed**

In our study, the checklist item regarding loss to follow-up does not apply as our analysis focused solely on short-term outcomes. There were no long-term follow-ups involved in the scope of this research.

**(e) Describe any sensitivity analyses**

Further details are provided in S3.

**13 (a) Report numbers of individuals at each stage of study—eg numbers potentially eligible, examined for eligibility, confirmed eligible, included in the study, completing follow-up, and analysed**

Page 11. Fig.1

**(b) Give reasons for non-participation at each stage**

Page 11. Fig.1

**(c) Consider use of a flow diagram**

Page 11. Fig.1

**14(a) Give characteristics of study participants (eg demographic, clinical, social) and information on exposures and potential confounders**

Page 10-11. Participant characteristics and details on exposures and confounders are comprehensively detailed in Table 1, providing a complete overview of demographic, clinical, and social factors, facilitating an understanding of the study's baseline data.

**(b) Indicate number of participants with missing data for each variable of interest**

Three participants with substantial missing data were excluded from the analysis. No missing data were present among the remaining participants.

**(c) Summarise follow-up time (eg, average and total amount)**

In our study, the checklist item regarding loss to follow-up does not apply as our analysis focused solely on short-term outcomes. There were no long-term follow-ups involved in the scope of this research.

**15 Report numbers of outcome events or summary measures over time**

Page 10-14. We reported the numbers of outcome events and summary measures over time through detailed tables. Table 1 shows baseline characteristics and covariate balance. Table 2 presents key intraoperative indicators. Table 3 compares postoperative outcomes.

**16 (a) Give unadjusted estimates and, if applicable, confounder-adjusted estimates and their precision (eg, 95% confidence interval). Make clear which confounders were adjusted for and why they were included**

Page 10-14. Our study provides both unadjusted and confounder-adjusted estimates. Baseline characteristics and outcomes before and after PSM are reported in the tables. This approach ensures clarity in how confounders were adjusted for and allows for comparison of estimates before and after adjustment, thereby meeting the checklist requirements.

Page 7. “Building on clinical expertise and prior research findings, we hypothesize that these indicators may influence the recovery process and the occurrence of postoperative complications in surgical patients. Therefore, these variables were adjusted for as confounding factors during the Propensity Score Matching (PSM) process.**”**

**(b) Report category boundaries when continuous variables were categorized**

Page 7. “The number of cysts was divided into two groups: equal to 1 and greater than 1.**” “**Abdominal drainage time was categorized into two groups: less than or equal to 7 days and greater than 7 days”

**(c) If relevant, consider translating estimates of relative risk into absolute risk for a meaningful time period**

This study is not relevant to converting relative risk estimates into absolute risk.

**17 Report other analyses done—eg analyses of subgroups and interactions, and sensitivity analyses**

We have already explained in item 12 that no additional subgroup analyses or interaction analyses were conducted.

**18 Summarise key results with reference to study objectives**

Page 14. In the first section of our discussion, we provided a description of the study objectives and key findings.

**19 Discuss limitations of the study, taking into account sources of potential bias or imprecision. Discuss both direction and magnitude of any potential bias**

Page 19. We described the primary sources of bias and the methods employed to reduce bias.

**20 Give a cautious overall interpretation of results considering objectives, limitations, multiplicity of analyses, results from similar studies, and other relevant evidence**

Page 20. Considering the study objectives, limitations, and relevant literature evidence, we cautiously provide our conclusions.

**21 Discuss the generalisability (external validity) of the study results**

Page 19. “Selection bias arises from the decisions made by doctors to choose different treatment options for different patients, while information bias stems from potential errors in medical record documentation. Although we used PSM to reduce selection bias, it cannot eliminate them entirely. There were significant differences in cyst diameter and location between the excluded and non-excluded patients (P < 0.05), which may limit the generalizability of the study results to patients with larger cysts.**”**

**22 Give the source of funding and the role of the funders for the present study and, if applicable, for the original study on which the present article is based**

I have already included a description of the sources of research funding and the role of sponsoring organizations in the study in the manuscript.
