# Supplementary material for: Perioperative outcomes and hospitalization costs of radical vs. conservative surgery for hepatic cystic echinococcosis: A retrospective study
Source: PLoS Negl Trop Dis. 2024 Nov 13;18(11):e0012620. doi: 10.1371/journal.pntd.0012620 (PMC11559981; doi:10.1371/journal.pntd.0012620)
Supplement: S1 Table — (DOCX) [file pntd.0012620.s004.docx]

**Sensitivity analysis through adjustment of different caliper values and matching strategies**

Table 1: Baseline covariates before and after matching (caliper value 0.2 and nearest matching strategy)

| Variables | Level | Before Matching | | |  | After Matching | | |
| --- | --- | --- | --- | --- | --- | --- | --- | --- |
|  |  | No | Yes | SMD^△^ |  | No | Yes | SMD^△^ |
| n |  | 324 | 110 |  |  | 191 | 103 |  |
| Age (mean (SD)) |  | 36.22 (17.77) | 35.63 (19.60) | -0.030 |  | 35.24 (17.97) | 35.07 (19.65) | -0.027 |
| Gender (%) | Male | 136 (42.0) | 52 (47.3) | 0.106 |  | 81(42.4) | 48 (46.6) | 0.058 |
|  | Female | 188 (58.0) | 58 (52.7) | -0.106 |  | 110 (57.6) | 55 (53.4) | -0.058 |
| Cyst location (%) | Left lobe | 58 (17.9) | 32 (29.1) | 0.246 |  | 49 (25.7) | 28 (27.7) | -0.021 |
|  | Right lobe | 234 (72.2) | 65 (59.1) | -0.267 |  | 117 (61.3) | 62 (60.2) | 0.030 |
|  | Both the lobes | 32 (9.9) | 13 (11.8) | 0.060 |  | 25 (13.1) | 13 (12.6) | -0.015 |
| Cyst diameter (cm, mean (SD)) |  | 10.36 (3.65) | 9.30 (3.57) | -0.297 |  | 9.62 (3.24) | 9.37 (3.62) | -0.044 |
| Number of cysts (%) | 1 | 231 (71.3) | 78 (70.9) | -0.009 |  | 132 (69.1) | 73 (70.9) | 0.011 |
|  | ＞1 | 93 (28.7) | 32 (29.1) | 0.009 |  | 59 (30.9) | 30 (29.1) | -0.011 |
| WHO type of cysts (%) | Type 1 | 155 (47.8) | 46 (41.8) | -0.122 |  | 81 (42.4) | 44 (42.7) | 0.010 |
|  | Type 2 | 50 (15.4) | 25 (22.7) | 0.174 |  | 39 (20.4) | 24 (23.3) | 0.035 |
|  | Type 3 | 54 (16.7) | 19 (17.3) | 0.016 |  | 34 (17.8) | 17 (16.5) | -0.013 |
|  | Type 4 | 65 (20.1) | 18 (16.4) | -0.100 |  | 37 (19.4) | 18 (17.5) | -0.039 |
|  | Type 5 | 0 (0.0) | 2 (1.8) | 0.136 |  | 0 (0.0) | 0 (0.0) | 0.000 |
| Epigastric pain (%) | Yes | 183 (56.5) | 64 (58.2) | 0.034 |  | 110(57.6) | 60 (58.3) | 0.000 |
|  | No | 141 (43.5) | 46 (41.8) | -0.034 |  | 81 (42.4) | 43 (41.7) | -0.000 |
| Abdominal mass (%) | Yes | 166 (51.2) | 62 (56.4) | 0.103 |  | 106 (55.5) | 58 (56.3) | 0.000 |
|  | No | 158 (48.8) | 48 (43.6) | -0.103 |  | 85 (44.5) | 45 (43.7) | -0.000 |
| History of abdominal surgery (%) | Yes | 59 (18.2) | 25 (22.7) | 0.108 |  | 44 (23.0) | 21 (20.4) | -0.070 |
|  | No | 265 (81.8) | 85 (77.3) | -0.108 |  | 147 (77.0) | 82 (79.6) | 0.070 |
| Fever (%) | Yes | 3 (0.9) | 1 (0.9) | -0.002 |  | 2 (1.0) | 1 (1.0) | -0.000 |
|  | No | 321 (99.1) | 109 (99.1) | 0.002 |  | 189 (99.0) | 102 (99.0) | 0.000 |
| Extrahepatic  cyst (%) | Yes | 29 (9.0) | 10 (9.1) | 0.005 |  | 17 (8.9) | 8 (7.8) | -0.034 |
|  | No | 295 (91.0) | 100 (90.9) | -0.005 |  | 174 (91.1) | 95 (92.2) | 0.034 |
| Open abdominal surgery  (%) | Yes | 317 (97.8) | 101 (91.8) | -0.220 |  | 185 (96.9) | 99 (96.1) | 0.018 |
|  | No | 7 (2.2) | 9 (8.2) | 0.220 |  | 6 (3.1) | 4 (3.9) | -0.018 |
| ^△^Standardized Mean Difference | | | | | | | | |

Table 2: Intraoperative indicators before and after matching (caliper value 0.2 and nearest matching strategy)

|  | Before Matching | | |  |  | After Matching | | |
| --- | --- | --- | --- | --- | --- | --- | --- | --- |
|  | CS | RS | P |  | CS | RS | Mean difference (95%CI) | P |
| n | 324 | 110 |  |  | 191 | 103 |  |  |
| Operative time (min, Mean (SD)) | 135.8(48.77) | 177.2(84.47) | ＜0.001 |  | 135.5（51.56） | 176.3（85.62） | 41.49 (28.55,54.43) | ＜0.001 |
| Blood loss (ml, Median (IQR)) | 50(50, 150) | 100 (50,200) | ＜0.001 |  | 50 (50,100) | 100 (50,200) | 50 (22.56, 50) | ＜0.001 |
| Blood transfusion (ml, Median (IQR)) | 0（0, 0） | 0（0, 0） | ＜0.001 |  | 0（0, 0） | 0（0, 0） | 0 (0,0) | ＜0.001 |

Table 3: Immediate perioperative outcomes before and after matching (caliper value 0.2 and nearest matching strategy)

|  | Before Matching | | |  |  |  | After Matching | | |
| --- | --- | --- | --- | --- | --- | --- | --- | --- | --- |
|  | Conservative surgery | Radical surgery | P |  | Conservative surgery | Radical surgery | Risk Ratio (95% CI) | Odds Ratio (95% CI) | P |
| n | 324 | 110 |  |  | 191 | 103 |  |  |  |
| Overall morbidity (%) | 100 (31%) | 35 (32%) | 0.85 |  | 59 (31%) | 33 (32%) | 1.04 (0.73-1.48) | 1.05 (0.63-1.77) | 0.84 |
| Bile leakage (%) | 36 (11%) | 6 (5.5%) | 0.083 |  | 179 (9.9%) | 6 (5.8%) | 0.59 (0.24-1.42) | 0.56 (0.22-1.45) | 0.23 |
| Cavity effusion (%) | 65(20%) | 27(25%) | 0.32 |  | 38(21%) | 25(25%) | 1.19 (0.76-1.85) | 1.25 (0.71-2.21) | 0.48 |
| Pulmonary infection (%) | 16(4.9%) | 4(3.6%) | 0.57 |  | 7(3.7%) | 3(2.9%) | 0.79 (0.21-3.01) | 0.79 (0.20-3.12) | ＞0.99 |
| Death (%) | 0(0%) | 1(0.9%) | 0.25 |  | 0(0%) | 1(1.0%) | - | - | 0.35 |
| ICU stay (%) | 8(2.5%) | 4(3.6%) | 0.51 |  | 3(1.6%) | 4(3.9%) | 2.47 (0.56-10.84) | 2.53 (0.56-11.54) | 0.24 |
| Abdominal drainage time > 7 days (%) | 201(62%) | 52(47%) | 0.007 |  | 107(56%) | 46(45%) | 0.80 (0.62-1.02) | 0.63 (0.39-1.03) | 0.063 |
| Postoperative hospital stays (Day Mean (SD)) | 9.4 (3.21) | 8.9 (2.50) | 0.119 |  | 9.2(3.07) | 8.9(2.54) | -0.27 (-0.96, 0.43) * | | 0.45 |
| Incision infection (%) | 1(0.3%) | 0(0%) | ＞0.99 |  | 1 (0.5%) | 0(0%) | - | - | ＞0.99 |
| Intestinal obstruction (%) | 1(0.3%) | 0(0%) | ＞0.99 |  | 0 (0%) | 0(0%) | - | - | ＞0.99 |
| Surgery cost (CNY^△^, mean (SD)) | 4768.6 (1752.83) | 5698.5 (1980.24) | ＜0.001 |  | 4837.5 (1902.96) | 5711.8 (2017.88) | 861.52 (395.27, 1327.78) * | | ＜0.001 |
| Total cost (CNY^△^, mean (SD)) | 21441.5 (6425.59) | 24206.8 (8940.21) | ＜0.001 |  | 21263.9 (6881.29) | 23994.6 (8825.44) | 2730.63 (905.56, 4555.70) * | | 0.004 |

△Chinese Yuan; * Mean Difference (95%CI)

Table 1: Baseline covariates before and after matching（caliper value 0.05 and nearest matching strategy）

| Variables | Level | Before Matching | | |  | After Matching | | |
| --- | --- | --- | --- | --- | --- | --- | --- | --- |
|  |  | No | Yes | SMD^△^ |  | No | Yes | SMD^△^ |
| n |  | 324 | 110 |  |  | 164 | 99 |  |
| Age (mean (SD)) |  | 36.22 (17.77) | 35.63 (19.60) | -0.030 |  | 34.93 (17.75) | 34.80 (19.87) | 0.002 |
| Gender (%) | Male | 136 (42.0) | 52 (47.3) | 0.106 |  | 67 (40.9) | 47 (47.5) | 0.121 |
|  | Female | 188 (58.0) | 58 (52.7) | -0.106 |  | 97 (59.1) | 52 (52.5) | -0.121 |
| Cyst location (%) | Left lobe | 58 (17.9) | 32 (29.1) | 0.246 |  | 40 (24.4) | 25 (25.3) | 0.000 |
|  | Right lobe | 234 (72.2) | 65 (59.1) | -0.267 |  | 100 (61.0) | 61 (61.6) | 0.031 |
|  | Both the lobes | 32 (9.9) | 13 (11.8) | 0.060 |  | 24 (14.6) | 13 (13.1) | -0.047 |
| Cyst diameter (cm, mean (SD)) |  | 10.36 (3.65) | 9.30 (3.57) | -0.297 |  | 9.56 (3.09) | 9.33 (3.60) | -0.057 |
| Number of cysts (%) | 1 | 231 (71.3) | 78 (70.9) | -0.009 |  | 111 (67.7) | 69 (69.7) | 0.011 |
|  | ＞1 | 93 (28.7) | 32 (29.1) | 0.009 |  | 53 (32.3) | 30 (30.3) | -0.011 |
| WHO type of cysts (%) | Type 1 | 155 (47.8) | 46 (41.8) | -0.122 |  | 69 (42.1) | 42 (42.4) | 0.051 |
|  | Type 2 | 50 (15.4) | 25 (22.7) | 0.174 |  | 31 (18.9) | 23 (23.2) | -0.000 |
|  | Type 3 | 54 (16.7) | 19 (17.3) | 0.016 |  | 32 (19.5) | 16 (16.2) | -0.094 |
|  | Type 4 | 65 (20.1) | 18 (16.4) | -0.100 |  | 32 (19.5) | 18 (18.2) | 0.027 |
|  | Type 5 | 0 (0.0) | 2 (1.8) | 0.136 |  | 0 (0.0) | 0 (0.0) | 0.000 |
| Epigastric pain (%) | Yes | 183 (56.5) | 64 (58.2) | 0.034 |  | 93 (56.7) | 58 (58.6) | 0.020 |
|  | No | 141 (43.5) | 46 (41.8) | -0.034 |  | 71 (43.3) | 41 (41.4) | -0.020 |
| Abdominal mass (%) | Yes | 166 (51.2) | 62 (56.4) | 0.103 |  | 90 (54.9) | 55 (55.6) | 0.000 |
|  | No | 158 (48.8) | 48 (43.6) | -0.103 |  | 74 (45.1) | 44 (44.4) | 0.000 |
| History of abdominal surgery (%) | Yes | 59 (18.2) | 25 (22.7) | 0.108 |  | 38 (23.2) | 20 (20.2) | -0.096 |
|  | No | 265 (81.8) | 85 (77.3) | -0.108 |  | 126 (76.8) | 79 (79.8) | 0.096 |
| Fever (%) | Yes | 3 (0.9) | 1 (0.9) | -0.002 |  | 2 (1.2) | 1 (1.0) | 0.000 |
|  | No | 321 (99.1) | 109 (99.1) | 0.002 |  | 162 (98.8) | 98 (99.0) | 0.000 |
| Extrahepatic  cyst (%) | Yes | 29 (9.0) | 10 (9.1) | 0.005 |  | 15 (9.1) | 8 (8.1) | -0.035 |
|  | No | 295 (91.0) | 100 (90.9) | -0.005 |  | 149 (90.9) | 91 (91.9) | 0.035 |
| Open abdominal surgery  (%) | Yes | 317 (97.8) | 101 (91.8) | -0.220 |  | 158 (96.3) | 96 (97.0) | 0.037 |
|  | No | 7 (2.2) | 9 (8.2) | 0.220 |  | 6 (3.7) | 3 (3.0) | -0.037 |
| ^△^Standardized Mean Difference | | | | | | | | |

Table 2: Intraoperative indicators before and after matching（caliper value 0.05 and nearest matching strategy）

|  | Before Matching | | |  |  | After Matching | | | |
| --- | --- | --- | --- | --- | --- | --- | --- | --- | --- |
|  | CS | RS | P |  | CS | RS | Mean difference (95%CI) | | P |
| n | 324 | 110 |  |  | 164 | 99 |  | |  |
| Operative time (min, Mean (SD)) | 135.8(48.77) | 177.2(84.47) | ＜0.001 |  | 133.3（48.23） | 176.2（81.36） | 42.84 (27.19, 58.49) | | ＜0.001 |
| Blood loss (ml, Median (IQR)) | 50(50, 150) | 100 (50,200) | ＜0.001 |  | 50 (50, 100) | 100 (50, 200) | | 50 (25.06, 50) | ＜0.001 |
| Blood transfusion (ml, Median (IQR)) | 0（0, 0） | 0（0, 0） | ＜0.001 |  | 0 (0,0) | 0 (0,0) | | 0 (0,0) | ＜0.001 |

Table 3: Immediate perioperative outcomes before and after matching（caliper value 0.05 and nearest matching strategy）

|  | Before Matching | | |  |  |  | After Matching | | |
| --- | --- | --- | --- | --- | --- | --- | --- | --- | --- |
|  | CS | RS | P |  | CS | RS | Risk Ratio (95% CI) | Odds Ratio (95% CI) | P |
| n | 324 | 110 |  |  | 164 | 99 |  |  |  |
| Overall morbidity (%) | 100 (31%) | 35 (32%) | 0.85 |  | 48 (29%) | 33(33%) | 1.14 (0.79-1.64) | 1.21 (0.71-2.07) | 0.49 |
| Bile leakage (%) | 36 (11%) | 6 (5.5%) | 0.083 |  | 16 (9.8%) | 6 (6.1%) | 0.62 (0.25-1.53) | 0.60 (0.23-1.58) | 0.29 |
| Effusion (%) | 65(20%) | 27(25%) | 0.32 |  | 31 (19%) | 25 (25%) | 1.34 (0.84-2.12) | 1.45 (0.80-2.64) | 0.22 |
| Pulmonary infection (%) | 16(4.9%) | 4(3.6%) | 0.57 |  | 6 (3.7%) | 3 (3.0%) | 0.83 (0.21-3.24) | 0.82 ( 0.20-3.37) | ＞0.99 |
| Death (%) | 0(0%) | 1(0.9%) | 0.25 |  | 0(0%) | 1(1.0%) | - | - | 0.38 |
| ICU stay (%) | 8(2.5%) | 4(3.6%) | 0.51 |  | 3(1.8%) | 4(3.0%) | 1.66 (0.34-8.05) | 1.68 (0.33-8.47) | 0.68 |
| Abdominal drainage time > 7 days (%) | 201(62%) | 52(47%) | 0.007 |  | 91 (55%) | 44 (44%) | 0.80 (0.62-1.04) | 0.64 (0.39-1.06) | 0.083 |
| Postoperative hospital stays (day mean (SD)) | 9.4 (3.21) | 8.9 (2.50) | 0.119 |  | 9.0 (2,72) | 8.9 (2.52) | -0.11 (-0.77, 0.55) * | | 0.755 |
| Incision infection (%) | 1(0.3%) | 0(0%) | ＞0.99 |  | 1(0.5%) | 0(0%) | - | - | ＞0.99 |
| Intestinal obstruction (%) | 1(0.3%) | 0(0%) | ＞0.99 |  | 0 (0%) | 0(0%) | - | - | ＞0.99 |
| Surgery cost (CNY△, mean (SD)) | 4768.6 (1752.83) | 5698.5 (1980.24) | ＜0.001 |  | 4909.3 (1961.50) | 5709.1 (2016.23) | 799.86 (305.38, 1294.34) * | | 0.002 |
| Total cost (CNY, mean (SD)) | 21441.5 (6425.59) | 24206.8 (8940.21) | ＜0.001 |  | 21370.7 (7103.21) | 23815.1 (8335.67) | 2444.45 (551.21, 4337.69) * | | 0.012 |

△Chinese Yuan; * Mean Difference (95%CI)

Table 1: Baseline covariates before and after matching (optimal matching strategy)

| Variables | Level | Before Matching | | |  | After Matching | | |
| --- | --- | --- | --- | --- | --- | --- | --- | --- |
|  |  | No | Yes | SMD^△^ |  | No | Yes | SMD^△^ |
| n |  | 324 | 110 |  |  | 220 | 110 |  |
| Age (mean (SD)) |  | 36.22 (17.77) | 35.63 (19.60) | -0.030 |  | 35.86 (18.06) | 35.63 (19.60) | -0.012 |
| Gender (%) | Male | 136 (42.0) | 52 (47.3) | 0.106 |  | 99 (45.0) | 52 (47.3) | 0.046 |
|  | Female | 188 (58.0) | 58 (52.7) | -0.106 |  | 121 (55.0) | 58 (52.7) | -0.046 |
| Cyst location (%) | Left lobe | 58 (17.9) | 32 (29.1) | 0.246 |  | 54 (24.5) | 32 (29.1) | 0.100 |
|  | Right lobe | 234 (72.2) | 65 (59.1) | -0.267 |  | 136 (61.8) | 65 (59.1) | -0.055 |
|  | Both the lobes | 32 (9.9) | 13 (11.8) | 0.060 |  | 30 (13.6) | 13 (11.8) | -0.056 |
| Cyst diameter (cm, mean (SD)) |  | 10.36 (3.65) | 9.30 (3.57) | -0.297 |  | 9.74 (3.34) | 9.30 (3.57) | -0.125 |
| Number of cysts (%) | 1 | 231 (71.3) | 78 (70.9) | -0.009 |  | 151 (68.6) | 78 (70.9) | 0.050 |
|  | ＞1 | 93 (28.7) | 32 (29.1) | 0.009 |  | 69 (31.4) | 32 (29.1) | -0.050 |
| WHO type of cysts (%) | Type 1 | 155 (47.8) | 46 (41.8) | -0.122 |  | 90 (40.9) | 46 (41.8) | 0.018 |
|  | Type 2 | 50 (15.4) | 25 (22.7) | 0.174 |  | 45 (20.5) | 25 (22.7) | 0.054 |
|  | Type 3 | 54 (16.7) | 19 (17.3) | 0.016 |  | 42 (19.1) | 19 (17.3) | -0.048 |
|  | Type 4 | 65 (20.1) | 18 (16.4) | -0.100 |  | 43 (19.5) | 18 (16.4) | -0.086 |
|  | Type 5 | 0 (0.0) | 2 (1.8) | 0.136 |  | 0 (0.0) | 2 (1.8) | 0.136 |
| Epigastric pain (%) | Yes | 183 (56.5) | 64 (58.2) | 0.034 |  | 126 (57.3) | 64 (58.2) | 0.018 |
|  | No | 141 (43.5) | 46 (41.8) | -0.034 |  | 94 (42.7) | 46 (41.8) | -0.018 |
| Abdominal mass (%) | Yes | 166 (51.2) | 62 (56.4) | 0.103 |  | 123 (55.9) | 62 (56.4) | 0.009 |
|  | No | 158 (48.8) | 48 (43.6) | -0.103 |  | 97 (44.1) | 48 (43.6) | -0.009 |
| History of abdominal surgery (%) | Yes | 59 (18.2) | 25 (22.7) | 0.108 |  | 49 (22.3) | 25 (22.7) | 0.011 |
|  | No | 265 (81.8) | 85 (77.3) | -0.108 |  | 171 (77.7) | 85 (77.3) | -0.011 |
| Fever (%) | Yes | 3 (0.9) | 1 (0.9) | -0.002 |  | 2 (0.9) | 1 (0.9) | 0.000 |
|  | No | 321 (99.1) | 109 (99.1) | 0.002 |  | 218 (99.1) | 109 (99.1) | 0.000 |
| Extrahepatic  cyst (%) | Yes | 29 (9.0) | 10 (9.1) | 0.005 |  | 18 (8.2) | 10 (9.1) | 0.032 |
|  | No | 295 (91.0) | 100 (90.9) | -0.005 |  | 202 (91.8) | 100 (90.9) | -0.032 |
| Open abdominal surgery  (%) | Yes | 317 (97.8) | 101 (91.8) | -0.220 |  | 213 (96.8) | 101 (91.8) | -0.182 |
|  | No | 7 (2.2) | 9 (8.2) | 0.220 |  | 7 (3.2) | 9 (8.2) | 0.182 |
| ^△^Standardized Mean Difference | | | | | | | | |

Table 2: Intraoperative indicators before and after matching (optimal matching strategy)

|  | Before Matching | | |  |  | After Matching | | | |
| --- | --- | --- | --- | --- | --- | --- | --- | --- | --- |
|  | CS | RS | P |  | CS | RS | Mean difference (95%CI) | | P |
| n | 324 | 110 |  |  | 220 | 110 |  | |  |
| Operative time (min, Mean (SD)) | 135.8(48.77) | 177.2(84.47) | ＜0.001 |  | 134.8 (51.74) | 177.2 (84.47) | 42.47 (27.71, 57.23) | | ＜0.001 |
| Blood loss (ml, Median (IQR)) | 50(50, 150) | 100 (50,200) | ＜0.001 |  | 50 (50, 100) | 100 (50, 200) | | 50 (30, 50) | ＜0.001 |
| Blood transfusion (ml, Median (IQR)) | 0（0, 0） | 0（0, 0） | ＜0.001 |  | 0 (0,0) | 0 (0,0) | 0 (0.0) | | ＜0.001 |

Table 3: Immediate perioperative outcomes before and after matching (optimal matching strategy)

|  | Before Matching | | |  |  |  | After Matching | | |
| --- | --- | --- | --- | --- | --- | --- | --- | --- | --- |
|  | CS | RS | P |  | CS | RS | Risk Ratio (95% CI) | Odds Ratio (95% CI) | P |
| n | 324 | 110 |  |  | 220 | 110 |  |  |  |
| Overall morbidity (%) | 100 (31%) | 35 (32%) | 0.85 |  | 67 (30%) | 35 (32%) | 1.04 (0.74-1.47) | 1.07 (0.65-1.75) | 0.8 |
| Bile leakage (%) | 36 (11%) | 6 (5.5%) | 0.083 |  | 24 (11%) | 6 (5.5%) | 0.5 (0.21-1.19) | 0.47 (0.19-1.19) | 0.1 |
| Effusion (%) | 65(20%) | 27(25%) | 0.32 |  | 43 (20%) | 27 (25%) | 1.26 (0.82-1.92) | 1.34 (0.77-2.31) | 0.29 |
| Pulmonary infection (%) | 16(4.9%) | 4(3.6%) | 0.57 |  | 9 (4.1%) | 4 (3.6%) | 0.89 (0.28-2.28) | 0.88 (0.27-2.94) | ＞0.99 |
| Death (%) | 0(0%) | 1(0.9%) | 0.25 |  | 0 (0%) | 1 (0.9%) | - | - | 0.33 |
| ICU stay (%) | 8(2.5%) | 4(3.6%) | 0.51 |  | 4 (1.8%) | 4 (3.6%) | 2 (0.51-7.85) | 2.04 (0.5-8.31) | 0.45 |
| Abdominal drainage time > 7 days (%) | 201(62%) | 52(47%) | 0.007 |  | 125 (57%) | 52 (47%) | 0.83 (0.66-1.05) | 0.68 (0.43-1.08) | 0.10 |
| Postoperative hospital stays (Day Mean (SD)) | 9.4 (3.21) | 8.9 (2.50) | 0.119 |  | 9.4 (3.37) | 8.9 (2.50) | -0.45 (-1.17, 0.26) * | | 0.21 |
| Incision infection (%) | 1(0.3%) | 0(0%) | ＞0.99 |  | 1 (0.5%) | 0 (0%) | - | - | ＞0.99 |
| Intestinal obstruction (%) | 1(0.3%) | 0(0%) | ＞0.99 |  | 0 (0%) | 0 (0%) | - | - | ＞0.99 |
| Surgery cost (CNY△, mean (SD)) | 4768.6 (1752.83) | 5698.5 (1980.24) | ＜0.001 |  | 4745.3 (1859.75) | 5698.5 (1980.24) | 953.16 (518.14, 1388.17) * | | ＜0.001 |
| Total cost (CNY, mean (SD)) | 21441.5 (6425.59) | 24206.8 (8940.21) | ＜0.001 |  | 21242.0 (6590.21) | 24206.8 (8940.21) | 2964.84 (1258.82, 4670.85) * | | ＜0.001 |

△Chinese Yuan; * Mean Difference (95%CI)
