# Supplementary material for: Perioperative outcomes and hospitalization costs of radical vs. conservative surgery for hepatic cystic echinococcosis: A retrospective study
Source: PLoS Negl Trop Dis. 2024 Nov 13;18(11):e0012620. doi: 10.1371/journal.pntd.0012620 (PMC11559981; doi:10.1371/journal.pntd.0012620)

**Table: Cost Comparison Between Radical Surgery (RS) and Conservative Surgery (CS) After Propensity Score Matching (PSM)**

|  | CS | RS | P* |
| --- | --- | --- | --- |
| Surgery-related Costs (median) | 9059.50 | 11194.00 | <0.001 |
| Non-surgery-related Costs (median) | 1058.50 | 998.50 | 0.107 |
| Consumables Costs (median) | 2749.20 | 3267.00 | 0.003 |
| Medication Costs (median) | 6843.79 | 7016.97 | 0.694 |
| Examination Costs (median) | 5117.40 | 5436.50 | 0.042 |
| Other Costs (Total) (median) | 1011.00 | 1009.50 | 0.982 |

*Kruskal-Wallis

**Figure: Comparison of Different Cost Categories Between Radical Surgery (RS) and Conservative Surgery (CS) After Propensity Score Matching (PSM)**


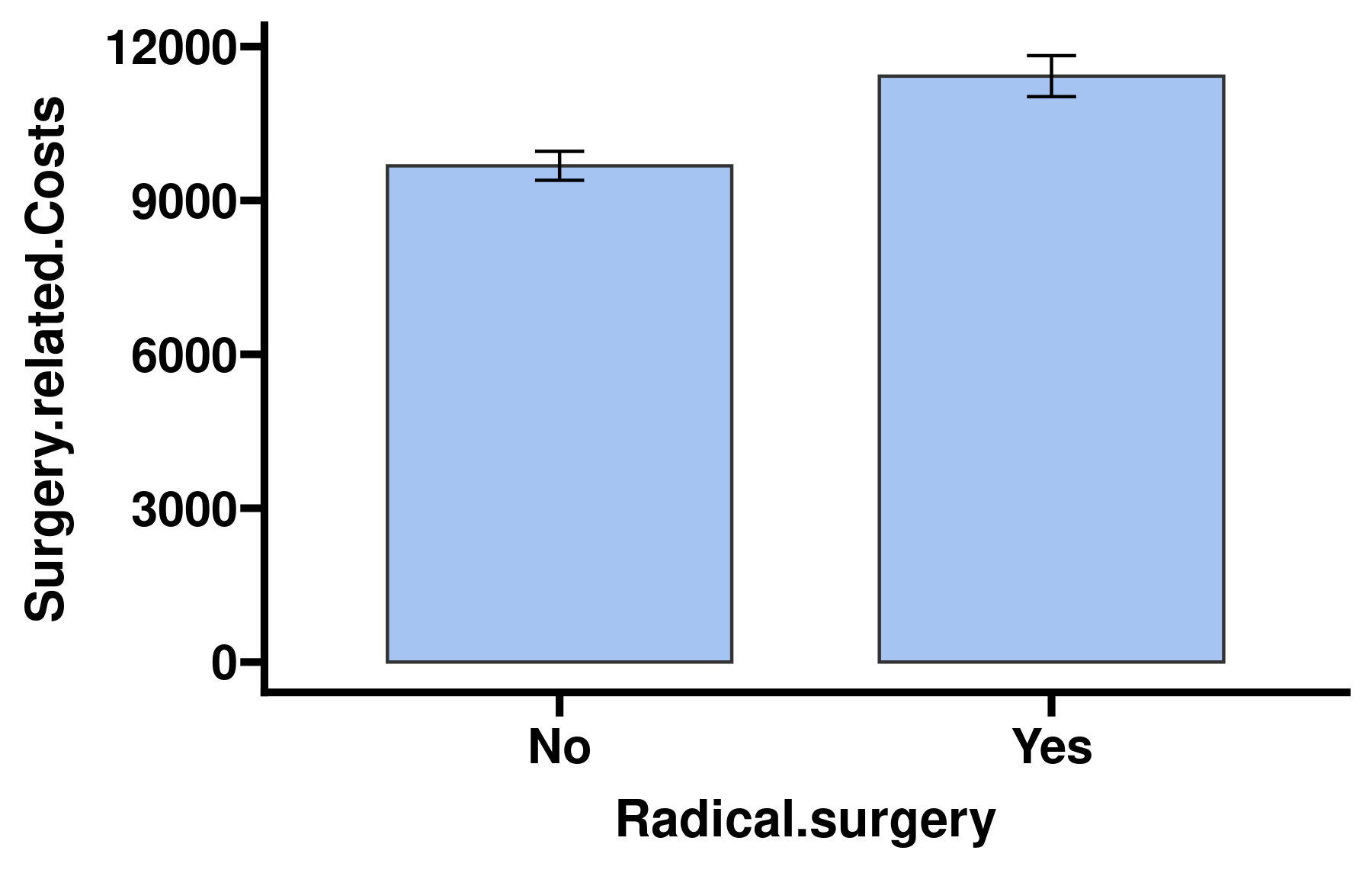

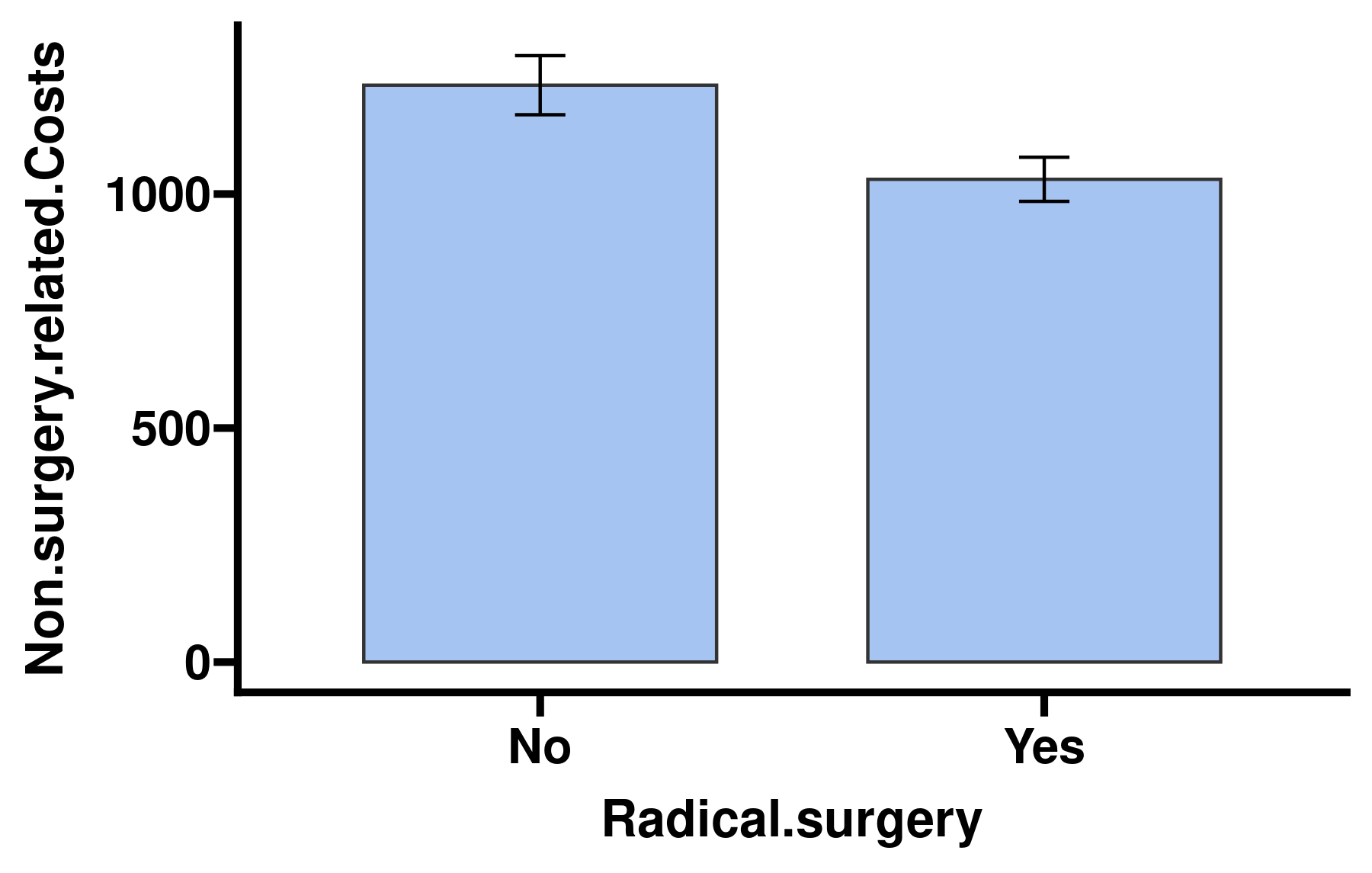


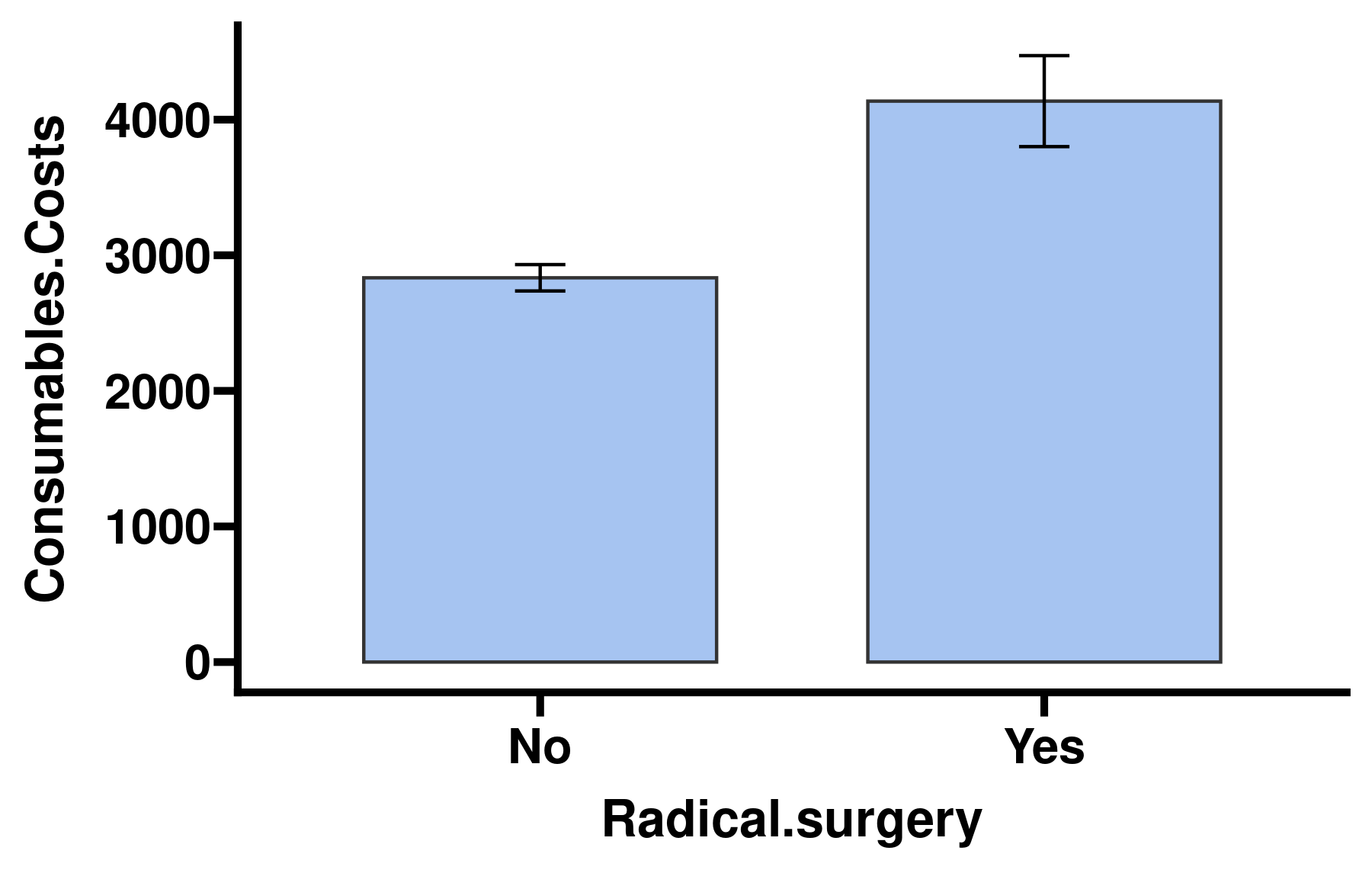

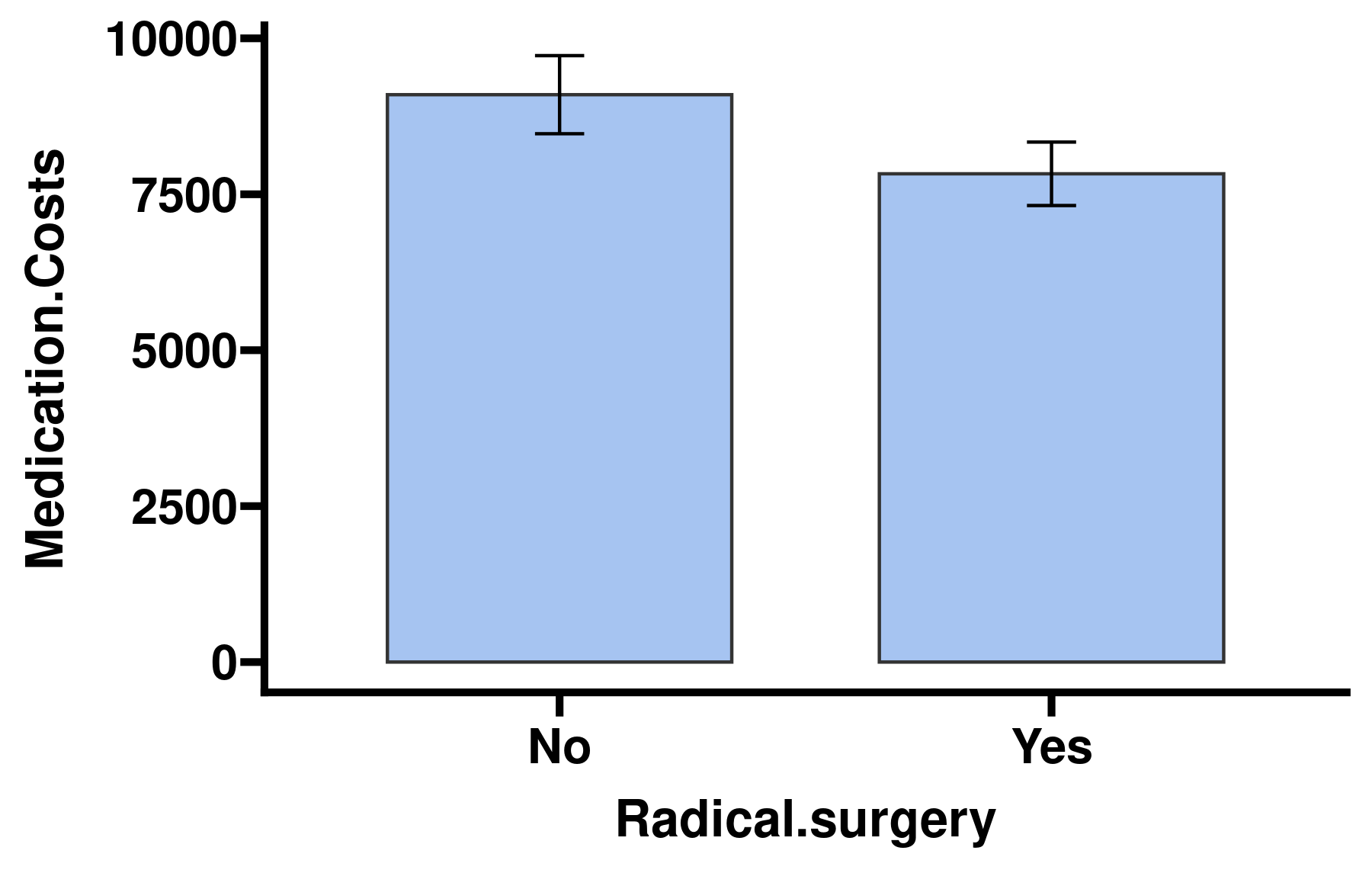


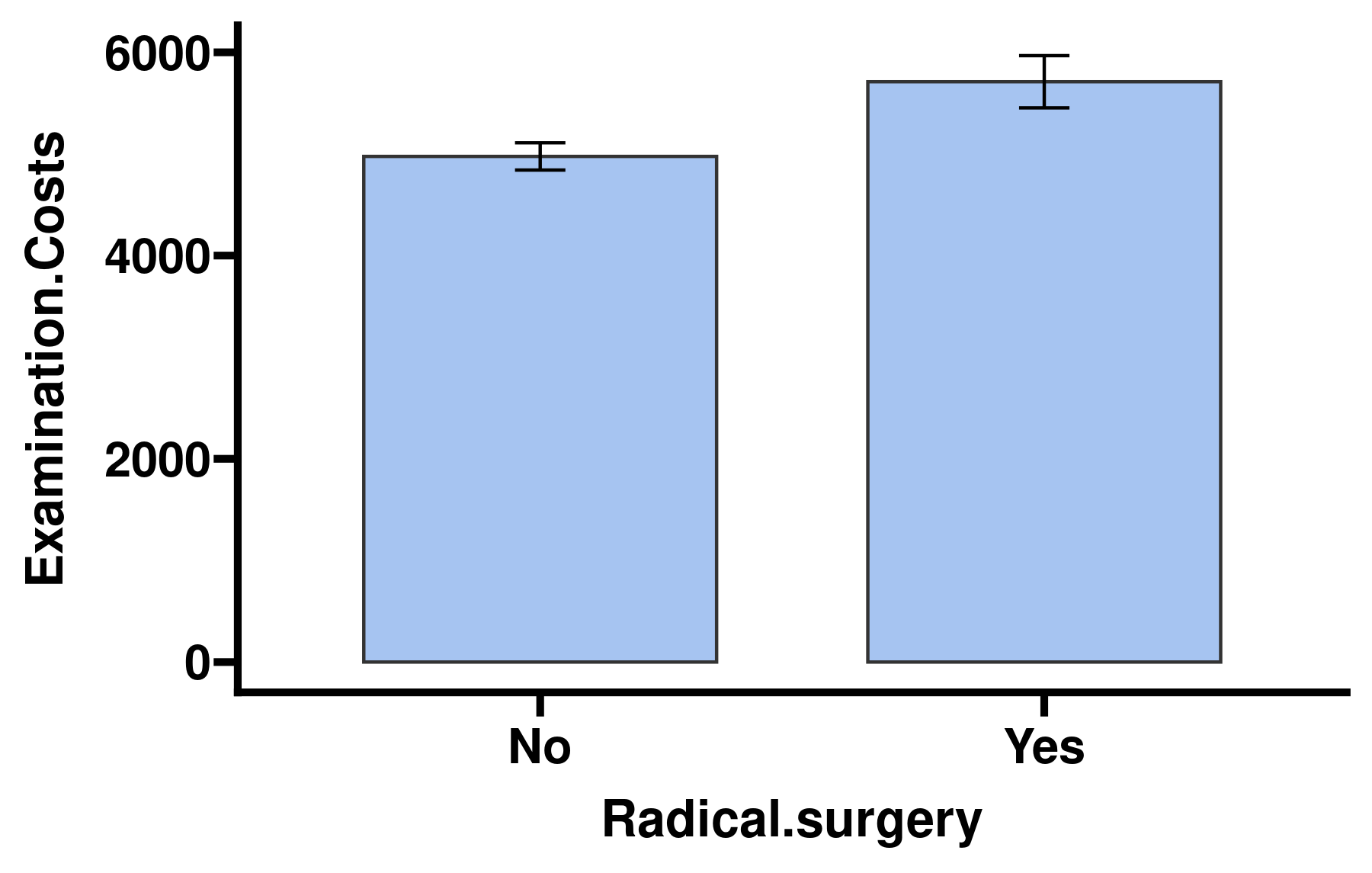

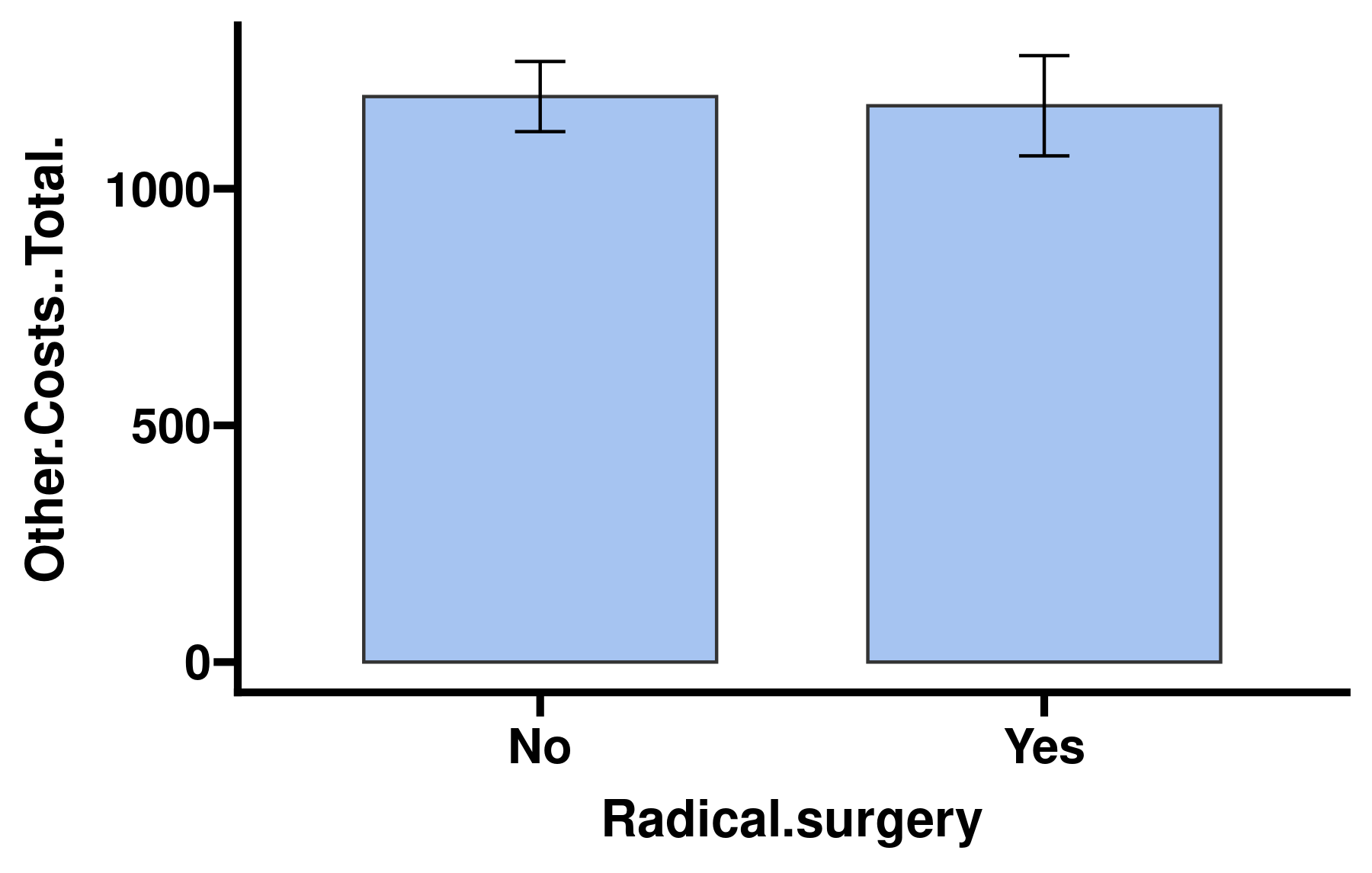

Supplement: S2 Table — (DOCX) [file pntd.0012620.s005.docx]
