# Supplementary material for: Perioperative outcomes and hospitalization costs of radical vs. conservative surgery for hepatic cystic echinococcosis: A retrospective study
Source: PLoS Negl Trop Dis. 2024 Nov 13;18(11):e0012620. doi: 10.1371/journal.pntd.0012620 (PMC11559981; doi:10.1371/journal.pntd.0012620)
Supplement: S3 Table — (DOCX) [file pntd.0012620.s006.docx]

**Table 1: Baseline covariates before and after matching**

| Variables | Level |  | Before Matching | | | |  |  | After Matching | | | |
| --- | --- | --- | --- | --- | --- | --- | --- | --- | --- | --- | --- | --- |
|  |  | No | | Yes | P | SMD^△^ |  | No | | Yes | P | SMD^△^ |
| n |  | 324 | | 110 |  |  |  | 182 | | 102 |  |  |
| Age (mean (SD)) |  | 36.22 (17.77) | | 35.63 (19.60) | 0.781 | -0.030 |  | 35.27 (18.02) | | 34.90 (19.67) | 0.877 | 0.015 |
| Gender (%) | Male | 136 (42.0) | | 52 (47.3) | 0.333 | 0.106 |  | 76 (41.8) | | 47 (46.1) | 0.481 | 0.088 |
|  | Female | 188 (58.0) | | 58 (52.7) |  | -0.106 |  | 106 (58.2) | | 55 (53.9) |  | -0.088 |
| Cyst location (%) | Left lobe | 58 (17.9) | | 32 (29.1) | 0.026 | 0.246 |  | 47 (25.8) | | 27 (26.5) | 0.971 | -0.022 |
|  | Right lobe | 234 (72.2) | | 65 (59.1) |  | -0.267 |  | 110 (60.4) | | 62 (60.8) |  | 0.050 |
|  | Both the lobes | 32 (9.9) | | 13 (11.8) |  | 0.060 |  | 25 (13.7) | | 13 (12.7) |  | -0.046 |
| Cyst diameter (cm, mean (SD)) |  | 10.36 (3.65) | | 9.30 (3.57) | 0.008 | -0.297 |  | 9.52 (3.09) | | 9.38 (3.64) | 0.734 | -0.039 |
| Number of cysts (%) | 1 | 231 (71.3) | | 78 (70.9) | 0.938 | -0.009 |  | 124 (68.1) | | 72 (70.6) | 0.668 | 0.054 |
|  | ＞1 | 93 (28.7) | | 32 (29.1) |  | 0.009 |  | 58 (31.9) | | 30 (29.4) |  | -0.054 |
| WHO cyst classification (%) | CE1 | 155 (47.8) | | 46 (41.8) | 0.068 | -0.122 |  | 77 (42.3) | | 43 (42.2) | 0.925 | -0.010 |
|  | CE2 | 50 (15.4) | | 25 (22.7) |  | 0.174 |  | 37 (20.3) | | 24 (23.5) |  | 0.023 |
|  | CE3a/b | 54 (16.7) | | 19 (17.3) |  | 0.016 |  | 33 (18.1) | | 17 (16.7) |  | -0.026 |
|  | CE4 | 65 (20.1) | | 18 (16.4) |  | -0.100 |  | 35 (19.2) | | 18 (17.6) |  | 0.013 |
|  | CE5 | 0 (0.0) | | 2 (1.8) |  | 0.136 |  | 0 (0.0) | | 0 (0.0) |  | 0.000 |
| Epigastric pain (%) | Yes | 183 (56.5) | | 64 (58.2) | 0.756 | 0.034 |  | 104 (57.1) | | 60 (58.8) | 0.783 | 0.010 |
|  | No | 141 (43.5) | | 46 (41.8) |  | -0.034 |  | 78 (42.9) | | 42 (41.2) |  | -0.010 |
| Abdominal mass (%) | Yes | 166 (51.2) | | 62 (56.4) | 0.352 | 0.103 |  | 103 (56.6) | | 58 (56.9) | 0.965 | -0.020 |
|  | No | 158 (48.8) | | 48 (43.6) |  | -0.103 |  | 79 (43.4) | | 44 (43.1) |  | 0.020 |
| History of abdominal surgery (%) | Yes | 59 (18.2) | | 25 (22.7) | 0.3 | 0.108 |  | 40 (22.0) | | 21 (20.6) | 0.784 | -0.023 |
|  | No | 265 (81.8) | | 85 (77.3) |  | -0.108 |  | 142 (78.0) | | 81 (79.4) |  | 0.023 |
| Fever (%) | Yes | 3 (0.9) | | 1 (0.9) | ＞0.999 | -0.002 |  | 2 (1.1) | | 1 (1.0) | ＞0.999 | 0.000 |
|  | No | 321 (99.1) | | 109 (99.1) |  | 0.002 |  | 180 (98.9) | | 101 (99.0) |  | 0.000 |
| Extrahepatic cyst (%) | Yes | 29 (9.0) | | 10 (9.1) | 0.965 | 0.005 |  | 17 (9.3) | | 8 (7.8) | 0.669 | -0.034 |
|  | No | 295 (91.0) | | 100 (90.9) |  | -0.005 |  | 165 (90.7) | | 94 (92.2) |  | 0.034 |
| Open abdominal surgery  (%) | Yes | 317 (97.8) | | 101 (91.8) | 0.007 | -0.220 |  | 176 (96.7) | | 99 (97.1) | ＞0.999 | 0.036 |
|  | No | 7 (2.2) | | 9 (8.2) |  | 0.220 |  | 6 (3.3) | | 3 (2.9) |  | -0.036 |
| ^△^Standardized Mean Difference | | | | | | | | | | | | |
