# Supplementary material for: Perioperative outcomes and hospitalization costs of radical vs. conservative surgery for hepatic cystic echinococcosis: A retrospective study
Source: PLoS Negl Trop Dis. 2024 Nov 13;18(11):e0012620. doi: 10.1371/journal.pntd.0012620 (PMC11559981; doi:10.1371/journal.pntd.0012620)
Supplement: Attachment — Submitted filename: Review (8).docx [file pntd.0012620.s007.docx]

In this retrospective study, the authors examine the differences in perioperative outcomes and costs between radical surgery (RS) and conservative surgery (CS) for the treatment of hepatic cystic echinococcosis. Using data from 434 patients treated at a single center, the study found that while RS is associated with longer operative times, greater blood loss, increased blood transfusions, and higher costs, it does not provide significant short-term clinical advantages over CS. The study concludes that CS may be a viable alternative, particularly in resource-limited settings.

The manuscript is generally well written with clear and concise language. The study addresses an important and ongoing debate in the surgical management of hepatic cystic echinococcosis and provides a thorough comparison of perioperative outcomes and costs between radical and conservative surgical approaches. The use of propensity score matching to mitigate selection bias is a strength and contributes to the robustness of the findings.

However, there are some important issues and questions which should be addressed:

1. The title is descriptive but can be shortened for clarity. Consider: "Perioperative Outcomes and Costs of Radical vs. Conservative Surgery for Hepatic Cystic Echinococcosis: A Retrospective Study".
2. How were surgical and total costs calculated? The method of calculation and cost details should be reported in Methods and results.
3. Please consider P-value for the result of statistical test in Table 1.
4. It is unclear, which factors (preoperative? Intraoperative? Etc.) were used for PSM. Since P-value was not reported in preoperative data (table 1), the rationality of PSM is questionable. A PSM itself can cause selection bias, when not indicated. Maybe it is better to report real-world data.

I recommend major revision before considering for publication.
