# Supplementary material for: Perioperative outcomes and hospitalization costs of radical vs. conservative surgery for hepatic cystic echinococcosis: A retrospective study
Source: PLoS Negl Trop Dis. 2024 Nov 13;18(11):e0012620. doi: 10.1371/journal.pntd.0012620 (PMC11559981; doi:10.1371/journal.pntd.0012620)
Supplement: Attachment — Submitted filename: Response Letter.docx [file pntd.0012620.s008.docx]

Dear Editor and Reviewers:

Thank you for reviewing our manuscript and providing constructive comments. Your feedback has been invaluable in helping us improving the manuscript. We have carefully considered all your suggestions and made significant revisions accordingly.

Additionally, we have made some minor revisions to improve readability without changing the original meaning, and these have been marked in the manuscript.

**Response to editor’s comments:**

Thank you for providing the detailed feedback. We appreciate the opportunity to further refine our manuscript based on your comments. Comments are numbered and listed below in blue font. Our replies are given in normal font. We made the revisions using the track changes mode.

**Major: Methods**

1) The cost assessment part is basically lacking. The authors must write the detailed methods for the cost estimation.

**Reply:** Thank you for bringing this to our attention. We acknowledge the lack of detailed methods for cost estimation in the manuscript. We have now expanded the Methods section to include a dedicated subsection on cost assessment at line 206-209. This subsection provides a clear description of the following:

Cost Components: We define the specific cost components included in our analysis, such as surgery-related costs, non-surgery-related costs, consumables costs, medication costs, examination costs, and other costs (Total).

Data Sources: We specify the source of the cost data, which is derived from patient admission records.

Calculation Methods: We outline the methods used to calculate the "total cost." For example, we explain how we extracted and aggregated the relevant cost information from the admission records.

For further clarity, we have also provided a detailed breakdown of the cost components and their definitions in S2, and a cost comparison between RS and CS after PSM in S5.

2) Data must be clearly presented regarding timing of data collection in relation to surgery. If more than one surgery was carried out during the period, it must be specified how data were handled. Info in S2 is insufficient. In S2 are also not specified important factors for surgical decision, such as location of the cyst (e.g. adjacent to a big vessel, superficial vs deep-seated cyst), which I would expect influence both the choice of surgery and possible complications. Please explain in the text why these factors were not considered or, if considered, how were handled.

**Reply:** 1. Thank you for your insightful comment. We appreciate the need for clarity regarding data collection timing. Our study employed a retrospective design, with data collection occurring at the time of patient discharge. To ensure data consistency and minimize potential confounding, we included only the *first* surgical procedure for each patient in the analysis. Patients who underwent multiple surgeries during the study period had subsequent procedures excluded. This approach was implemented to prevent bias from repeated data points and ensure that each patient contributed only once to the dataset, thereby maintaining the validity of our statistical comparisons between surgical groups.

2. We appreciate your valuable comments regarding the impact of cyst location on surgical decision-making. We recognize the importance of factors such as proximity to major vessels and the depth of the cyst. However, as this was a retrospective study, detailed anatomical information was not systematically recorded in our database. This represents a limitation of our study.

It is important to note that the choice between radical and conservative surgery was made by our hepatobiliary surgeons based on each patient's individual condition, in conjunction with their experience and local resources. While certain factors (e.g., proximity to major vessels, deep-seated cysts) were not quantified, they were considered during the actual surgical decision-making process. We included relevant variables in our analysis, such as cyst location, size, and WHO classification, to minimize bias in surgical decision-making.

We acknowledge the need for more detailed anatomical information in future studies. A prospective data collection approach would allow for the inclusion of such data and further refine surgical selection criteria.

3) line 133: “overall morbidity”. In S2 the definition is quite broad...please be more specific about complications included ad what "adverse outcomes" includes

**Reply:** Thank you for your suggestion. We apologize for the lack of clarity in our previous definition of "Overall morbidity." We have now revised the definition in S2 to provide greater specificity. The updated definition is: Overall morbidity (yes/no): including bile leak, effusion (postoperative pleural or abdominal effusion), pulmonary infection, death, ICU stay, abdominal drainage time days>7, incision infection, intestinal obstruction.

**Major: Results**

cyst classification (e.g. Table 1) The WHO-IWGE classification does not include “Type 1, Type 2, Type 3, Type 4 and Type 5” but CE1, CE2, CE3a, CE3b, CE4, CE4. Consider also that “CE3” per se does not exists, but there are CE3a (with detached parasitic layers) and CE3b (partially solid with daughter cysts). Please apply throughout the correct cyst classification.

**Reply:** Thank you for pointing out the error in our cyst classification. We have carefully reviewed the hospital records and reclassified the data according to the latest WHO-IWGE classification system. We have revised Table 1 to reflect this correction, ensuring that the correct cyst classifications are used throughout the manuscript.

**Major: Discussion**

1) in line 217 the authors state that there were no significant differences in short-term clinical outcomes between the groups, but later on (line 226), on the contrary, they state that radical surgery had “higher risks”. Please clarify

**Reply:** Thank you for your insightful feedback. We recognize that our previous wording created confusion regarding the comparison of short-term outcomes and the complexity of the surgical procedures.

In the original manuscript, at Line 226, we mistakenly used the word "risk" instead of "complexity." Our intended message was that radical surgery (RS) is more complex and technically demanding compared to conservative surgery (CS).

We have now rewritten the second paragraph of the Discussion section to clarify this point. We have also revised the sentence in the first paragraph to: " We found that RS is associated with greater surgical complexity, including longer operative times and increased blood loss, along with higher costs."

We apologize for any confusion caused by the previous wording and appreciate your attention to detail.

2) The limitations of the study should be more clearly indicated and highlighted. For example, in line 232: the authors mention “beyond recurrence rate”: this is not a secondary thing but a very strong limitation of this study because recurrence is a serious issue and often surgeons who do not follow the patients for a long time are completely unaware about the recurrences and think all their work was perfect, therefore this severe study limitation must be stressed as such. Also, despite the matching process, there are 150 excluded and this could have biased the results.
Please provide in the methods any possible way the EXCLUDED people may have been systematically excluded and/or different from the included patients and in the discussion stress this big number of excluded patients and its consequences also a major limitation of the study

**Reply:** Thank you for your valuable comments. We agree that the limitations of our study should be more clearly highlighted.

**Regarding the lack of long-term follow-up on recurrence rates:**

We acknowledge that the absence of long-term follow-up data on recurrence rates is a significant limitation of our study. This is a critical factor in evaluating the long-term success of surgical interventions for hydatid cysts. We have revised the manuscript to emphasize this limitation in the Discussion section. We have also added a sentence in the Limitations section to explicitly state that the lack of long-term follow-up data on recurrence rates could affect the interpretation of our findings.

**Regarding the exclusion of patients during the matching process:**

We appreciate your concern about the potential bias introduced by the exclusion of 150 patients during the propensity score matching (PSM) process. We used PSM to minimize confounding factors between RS and CS groups, aiming to improve the comparability of the results.

We have added a detailed explanation of the matching process and the characteristics of the excluded patients to the Methods section. This explanation clarifies that the excluded patients were primarily from the CS group and tended to have larger cysts. We have also included a statement in the Discussion section acknowledging that this exclusion may limit the generalizability of our findings to patients with larger cysts.

We recognize that the large number of excluded patients is a potential limitation. However, we believe that the PSM approach, while not perfect, helped to control for significant confounding factors and allowed us to draw meaningful conclusions about the effectiveness of the two surgical methods.

We acknowledge that future research should consider increasing the sample size and exploring alternative matching methods to further address these limitations and improve the generalizability of the findings. We have added a sentence in the Discussion section to emphasize the need for future research with larger sample sizes and more robust matching techniques.

3) Line 308-309 and 326-327: the sentences are completely unclear: why CS would be better than RS “considering the absence of data about recurrences”? What if all CS induce recurrence? Just with the data presented this cannot be so strongly concluded. Considering that this seems the core of the message from this study, this needs to be thoroughly clarified.

**Reply:** Thank you for your insightful comments. We understand your concerns regarding the comparison between CS and RS, especially in the absence of recurrence data.

Our intention was not to definitively state that CS is always better than RS, but rather to highlight that in resource-limited settings or for patients who are not ideal candidates for complex surgeries, CS may be a preferable option due to its lower operational complexity, despite the potential for recurrence.

We acknowledge that the absence of recurrence data limits our ability to fully assess the long-term outcomes of CS compared to RS. To address your concerns, we have revised the corresponding sections to clarify that our conclusions about CS being preferable in certain situations are based on short-term clinical outcomes and the feasibility of the procedure in resource-limited environments, rather than on long-term recurrence rates. We have also tempered our language to reflect the limitations of the available data. The revised text now reads: “Recurrence is a critical indicator of the efficacy of surgical interventions for HCE. One major limitation of this study is the lack of long-term follow-up data which could affect the interpretation of our findings.” “Future studies should include extended follow-up periods to comprehensively evaluate the long-term outcomes of different surgical approaches and to determine the most appropriate surgical method based on individual patient characteristics.”

We appreciate your attention to detail and hope this revised language more accurately reflects the limitations of our study and the nuances of the comparison between CS and RS.

4) Line 323-324: the authors seem implying that there are patients for which the risk of recurrence is warranted. This seems awkward to say the least. Please clarify what you mean

**Reply:** Thank you for pointing this out. We understand that the wording in Lines 323-324 might be misleading. What we intended to convey is not that recurrence risk is "warranted" for certain patients, but rather that in some cases, due to the limited resources or specific clinical conditions, CS might be chosen despite a potential recurrence risk.

We have revised this to clarify our intended meaning and avoid any misunderstanding. The revised sentence now in Conclusion Section reads: “This suggests that CS may be a viable alternative, particularly in resource-limited settings or for patients who are not suitable for more complex procedures. Further randomized controlled trials with long-term follow-up are needed to determine the optimal surgical approach for HCE.”

**OTHER:**
Abstract and line 67 and line 91-92: surgery is not the primary treatment for hepatic cystic echinococcosis. The WHO recommends a stage-specific approach for uncomplicated hepatic CE cysts, that takes also into consideration their size, and therefore cysts are recommended to be treated with albendazole alone (for example small CE1 or CE3a), percutaneous procedures (CE1, CE3a), or not treated at all and just followed-up over time (CE4-CE5). Please amend to convey a correct information

**Reply:** Thank you very much for your suggestion. We acknowledge that surgery is not always the primary treatment for hepatic cystic echinococcosis. We have carefully reviewed our manuscript and have made the changes to reflect the latest WHO recommendations for stage-specific management of HCE. We also have reorganized the data according to the latest WHO classification for echinococcosis and have revised **Table 1** accordingly.

Line 93-96: please use the current terminology for surgery (ref doi:10.1051/parasite/2020024.) here and throughout the text

**Reply:** Thank you for your suggestion. We have carefully reviewed our manuscript and have revised the text to use the current terminology for surgery, as recommended in the reference you provided (doi:10.1051/parasite/2020024).

Line 84: E granulosus in italic

**Reply:** Thank you for your correction. We have italicized it at Line 98 and throughout the manuscript to ensure consistent scientific nomenclature. We have also made sure to use the latest terminology for surgical procedures related to hepatic cystic echinococcosis, as recommended in the reference you provided (doi:10.1051/parasite/2020024).

Line 87: dissemination is not a symptom

**Reply:** Thank you for your valuable comment. We have revised the sentence accordingly to clarify that dissemination is not a symptom. The revised sentence now reads: "Ruptures of CE cysts can induce symptoms such as urticaria, fever, and even anaphylactic shock, and may also lead to dissemination of the parasite." This revision distinguishes the symptoms from the subsequent possible dissemination of the parasite.

Line 113: what is “color ultrasound”?

**Reply:** Thank you for your comment. We have revised the term "color ultrasound" to "Color Doppler Ultrasound" to better reflect the commonly used and accurate terminology. This change ensures clarity and consistency in our description of the imaging technique used in the study.

Line 114: what exact list of incomplete data would make the patient excluded from the analysis?

**Reply:** Thank you for your suggestion. We have revised the sentence in line XXX to clarify the term "incomplete medical records." The final version now reads: " Patients with incomplete medical records (specifically missing preoperative Color Doppler Ultrasound results, surgical records, or postoperative clinical course records) were excluded." This revision provides a more specific explanation of the exclusion criteria, ensuring transparency and clarity in our methodology.

Line 144: what does “internal cysts” means?

**Reply:** Thank you for your comment. The term "internal cysts" on line 144 was intended to refer to the internal layers of the cyst, specifically the adventitial, laminated, and germinal layers. To avoid ambiguity, we have revised the term to " most of the cyst layers " on line 243 for clarity. This change ensures a more precise and understandable description of the cyst's structure.

Line 219: the authors state that RS would provide more “comprehensive solution” for HCE. However, the authors did not investigate relapses, therefore they cannot drive this consideration from their study. If the drive it from other studies, they must be referenced. Please amend the sentence and clarify

**Reply:** Thank you for your excellent suggestion. We acknowledge that our study did not directly investigate recurrence rates, and therefore we cannot definitively conclude that RS provides a more comprehensive solution for HCE based solely on our findings. We have revised the language from lines 351 to 374 in the discussion section to clarify the differences between RS and CS and to highlight the significance of our study. We appreciate your attention to detail and hope these changes improve the accuracy and clarity of our discussion.

Line 222: what characteristics would have the patients “with a low risk of recurrence”?

**Reply:** Thank you for your suggestion. We acknowledge that our current study is not designed to identify patients with a low risk of recurrence. Therefore, we have deleted the sentence on line 222 regarding this aspect.

We plan to explore this important topic in future research, aiming to develop a better understanding of factors that contribute to recurrence and identify patients who may benefit from specific treatment strategies.

Line 338-340: please rephrase for clarity and explain the beaning of the biases you mention and in what direction such biases might have influenced your results

**Reply:**

Thank you for your suggestion. I have provided an explanation of the biases and further elaborated on how these biases limit the extrapolation of the results from 572-579.

**Response to reviewers’ comments:**

**Reviewer #1**

Comments for Line 44: "Surgical intervention is the primary treatment for hepatic cystic echinococcosis" and Line 90: "The primary treatment for hepatic cystic echinococcosis (HCE) involves surgical method". Please include in the abstract that today for uncomplicated hepatic CE and according to WHO classification and suggestions they are 4 options: medical treatment with albendazol; percutaneous; surgery (open or laparoscopic) and watch and wait. And for complicated CE hepatic cysts surgery is the treatment of choice.
**Reply:** Thank you for your professional correction. We appreciate your detailed feedback regarding the treatment options for HCE.

In the introduction, we have revised the sentence on line 152 from "The primary treatment for hepatic cystic echinococcosis (HCE) involves surgical methods, supplemented by percutaneous treatments and pharmacological interventions" to " Management of hepatic cystic echinococcosis (HCE) is complex and includes surgical treatments, percutaneous treatment, chemotherapy, and “watch and wait”". This revision reflects the current understanding of HCE management, acknowledging the various treatment modalities available.

We understand that the abstract has limited space and therefore focused on the surgical aspect of HCE treatment. We have revised the sentence on line 49 to " Surgical intervention is a crucial treatment for hepatic cystic echinococcosis. However, the choice between radical and conservative surgery remains controversial." This revision clarifies the importance of surgery in HCE management, particularly for complicated cases, while acknowledging the ongoing debate regarding the best surgical approach.

Line 146: Following surgery, patients in the CS group routinely received albendazole at a dosage of 10-15 mg/kg/day for three months. Comment: do you have the data of preoperative albendazole. If you got, please include it.
**Reply:** Thank you for your valuable suggestion. We appreciate your attention to detail. Unfortunately, we do not have data on the preoperative use of albendazole in our patients. This information was not systematically collected during the study period. We acknowledge that this limitation could potentially influence our results, as preoperative albendazole use might have affected the outcomes in both groups. We will consider incorporating this data collection in future studies to provide a more comprehensive understanding of the impact of albendazole on HCE treatment.

In line 299 and 329 and Fig 1, word "hydatid" is used. Please adjust the nomenclature according to: Vuitton DA, McManus DP, Rogan MT, Romig T, Gottstein B, Naidich A, Tuxun T, Wen H, Menezes da Silva A; World Association of Echinococcosis. International consensus on terminology to be used in the field of echinococcoses. Parasite. 2020;27:41. doi: 10.1051/parasite/2020024. Epub 2020 Jun 3. PMID: 32500855; PMCID: PMC7273836.
**Reply:** Thank you for pointing out the use of the term "hydatid" in our manuscript. We have carefully reviewed the latest nomenclature guidelines provided in the reference you suggested.

We have revised the term "hydatid" to "cystic" in lines 529 and Figure 1 to align with the recommended terminology. We appreciate your attention to detail and hope this revision improves the accuracy and consistency of our manuscript.

Table 1: WHO type 1/2/3/4. Please use appropriate classification: CE1/CE2/CE3a/CE3b/CE4/CE5. I noted that they are many cases CE4 cysts under surgical treatment. Why? Usually, CE4 type cysts can be managed with watch and wait strategies.
**Reply:** Thank you for your valuable comment. We have reviewed the hospital records again and reclassified the data according to the latest WHO classification system. Consequently, we have revised **Table 1** accordingly. Additionally, many of the CE4 patients in our study underwent surgery. This was partly due to the large size of the cysts, which caused clinical symptoms such as abdominal pain or palpable masses. Additionally, our hospital is located in a resource-limited area where consistent follow-up is challenging. This makes the "watch and wait" strategy less effective as many patients do not return for follow-up appointments. Therefore, after careful consideration and communication with the patients, we opted for surgery as a more proactive treatment approach in these cases.

Please include in the abstract that in regions with limited resource according to results, CS might be a more appropriate choice.
**Reply:** Thank you for your valuable suggestion. We agree that this sentence should be included to present the results more comprehensively. We have incorporated it into the conclusion of the abstract and the conclusion section as follows: "Conservative surgery may be a viable option in resource-limited settings or for patients unsuitable for complex procedures. Further research with long-term follow-up is needed to determine the optimal approach."

**Reviewer #2**

I just suggest to avoid use of Chinese currency to describe the costs of procedures. I would prefer a more international one, just to allow a better understanding
**Reply:** Thank you for your valuable suggestion. We understand the importance of using a more internationally recognized currency for clarity. We have revised Table 3 to reflect a more widely understood currency unit.

**Reviewer #3**

1. The title is descriptive but can be shortened for clarity. Consider: "Perioperative Outcomes and Costs of Radical vs. Conservative Surgery for Hepatic Cystic Echinococcosis: A Retrospective Study".

**Reply:** Thank you for your excellent suggestion. We agree that shortening the title improves clarity and readability. We have updated the manuscript title to: " Perioperative outcomes and hospitalization costs of radical vs. conservative surgery for hepatic cystic echinococcosis: A retrospective study"

2. How were surgical and total costs calculated? The method of calculation and cost details should be reported in Methods and results.

**Reply:** We have now expanded the Methods section to include a dedicated subsection on cost assessment at lines 206-209. We removed the 'surgical cost' category and instead conducted a new analysis of the 'total cost.' We extracted cost data for each patient from the hospital's admission records and categorized total cost into the following groups: surgery-related costs, non-surgery-related costs, consumables costs, medication costs, examination costs, and other costs (Total).

For further clarity, we have also provided a detailed breakdown of the cost components and their definitions in S2, along with a cost comparison between RS and CS after PSM in S5.

3. Please consider P-value for the result of statistical test in Table 1.

**Reply:** Thank you for your valuable suggestion. While both standardized mean difference (SMD) and p-values can be used to assess covariate balance in propensity score matching (PSM), p-values are sensitive to sample size and may not fully reflect post-matching balance. Therefore, we opted to present the more commonly used and stable SMD values in Table 1 to better illustrate the quality of our matching.

For your reference, we have included a supplementary table S6 that presents the p-values for each covariate. This table shows that after PSM, all SMD values are below 0.1, and p-values are greater than 0.05, indicating good covariate balance between the groups.

4. It is unclear, which factors (preoperative? Intraoperative? Etc.) were used for PSM. Since P-value was not reported in preoperative data (table 1), the rationality of PSM is questionable. A PSM itself can cause selection bias, when not indicated. Maybe it is better to report real-world data.

**Reply:** Thank you for your insightful comment. We apologize for the lack of clarity regarding the factors used for propensity score matching (PSM) in our original submission. We have made the following revisions to address your concerns:

1. Clarifying PSM Factors:

We have added a sentence in the Methods section to explicitly state the factors included in the PSM: "Therefore, we selected preoperative and intraoperative data as confounding factors during the propensity score matching (PSM). The preoperative data included age, gender, cyst location, cyst diameter, number of cysts, WHO cyst classification, epigastric pain, abdominal mass, fever, and extrahepatic cysts. The intraoperative factor was open abdominal surgery."

We have also revised the sentence in the Statistical Analysis section to clarify the variables used in the propensity score calculation: "Propensity scores were calculated using logistic regression, incorporating relevant preoperative demographic and clinical characteristics, as well as the intraoperative factor of open abdominal surgery."

2. Addressing P-value Concerns:

As previously mentioned, we have included a supplementary table S6 with p-values for your reference. This table shows that after PSM, all SMD values are below 0.1, and p-values are greater than 0.05, indicating good covariate balance between the groups.

3. Acknowledging Potential Bias:

We agree that PSM itself can introduce bias. Therefore, we have reported the outcomes and costs for the RS and CS groups both before and after matching for the readers' review. Except for abdominal drainage time, the other results are consistent.

We have also addressed the issue regarding abdominal drainage time in the Discussion section.

We appreciate your detailed suggestions and hope these revisions have improved the clarity and robustness of our study.
